# Implementing a Cardiology Quality Incentive Program to Improve Guideline-Directed Medical Therapy

**DOI:** 10.1016/j.jacadv.2025.101879

**Published:** 2025-06-20

**Authors:** David J. Cho, Pooya I. Bokhoor, Anna Dermenchyan, Nicholas Brownell, Nina Lou Delavin, Sean Furlong, Juyea Hoo, Tristan Tibbe, Lucia Y. Chen, Sitaram Vangala, Benjamin A. Waterman, Maria Han, Gregg C. Fonarow, Priscilla Y. Hsue, Chidinma Chima-Melton

**Affiliations:** a Department of Medicine, Division of Cardiology, UCLA Health, Los Angeles, California, United States; b Department of Medicine, Quality, UCLA Health, Los Angeles, California, United States; c UCLA Faculty Practice Group, Office of Population Health and Accountable Care, University of California, Los Angeles, California, United States; d Department of Medicine, Division of Internal Medicine, UCLA Health, Los Angeles, California, United States; e UCLA Department of Medicine Statistics Core, UCLA Health, Los Angeles, California, United States

**Keywords:** guideline-directed medical therapy, implementation science, pay for performance, physician incentive, quality improvement

## Abstract

**Background:**

Adherence to guideline-directed medical therapy (GDMT) is central to quality-improvement programs, although the impact of financial incentive programs has been mixed.

**Objectives:**

Assess the impact of a cardiovascular population health initiative that integrates financial incentives, robust data infrastructure, and electronic health record clinical decision support on improving GDMT for cardiovascular disease (CVD).

**Methods:**

The program was implemented across 15 ambulatory clinics with 54 cardiologists in an academic health system. Individualized CVD patient panels were created for each provider, and providers received quarterly performance and incentive reports. Quality metrics included antiplatelet and statin or proprotein convertase subtilisin/kexin type 9 inhibitor therapy for atherosclerotic cardiovascular disease prevention, blood pressure control, and GDMT for heart failure with reduced ejection fraction (HFrEF; specified beta blockers; ACEI, ARB, or ARNI; mineralocorticoid receptor antagonist). An interrupted time series analysis evaluated monthly, 1-year, and 2-year changes in the odds of adhering to each specific metric associated with the implementation of the cardiovascular population health program.

**Results:**

After the intervention, the composite HFrEF therapy metric improved significantly (2-year odds ratio [OR]: 2.285; 95% confidence interval [CI]: 1.653-3.158; *P* < 0.001). Individual metrics also improved, including mineralocorticoid receptor antagonist (2-year OR: 3.039; 95% CI: 2.520-3.663; *P* < 0.001); specified beta blockers (2-year OR: 1.430; 95% CI: 1.129-1.810; *P* = 0.003); angiotensin-converting enzyme inhibitor, angiotensin receptor blocker, or angiotensin receptor-neprilysin inhibitor therapy for HFrEF (2-year OR: 1.228; 95% CI: 1.001-1.505; *P* = 0.049); statin or proprotein convertase subtilisin/kexin type 9 inhibitor therapy for atherosclerotic cardiovascular disease (2-year OR: 1.146; 95% CI: 1.092-1.202; *P* < 0.001); and blood pressure control (2-year OR: 1.496; 95% CI: 1.444-1.550; *P* < 0.001).

**Conclusions:**

Our program was associated with sustained improvements in GDMT adherence for CVD. It may serve as a scalable model for enhancing the quality of cardiovascular care.

## Key Points

• Can a large academic health system successfully build a cardiology quality incentive program that improves and sustains adherence to guideline-directed medical therapies for cardiovascular disease and HFrEF?
• In this prospective observational study that included 54 cardiologists and 75,239 patients, composite outcome of HFrEF therapies; specified beta blockers; ACEI, ARB, or ARNI; and mineralocorticoid receptor antagonist therapies in HFrEF, LDL-lowering therapy for primary and secondary atherosclerotic cardiovascular disease prevention, and blood pressure control demonstrated improvements in the postintervention trend compared to preintervention.
• A cardiology quality-improvement program can be successfully designed and implemented to improve and sustain guideline-directed medical therapy performance for cardiovascular disease and HFrEF. Our program helped clinicians monitor and improve their individual performance while identifying areas of improvement and can serve as a model for the implementation of ambulatory cardiovascular quality programs on a broader scale.

Increasing adherence to guideline-directed medical therapy (GDMT) is a major focus of quality improvement (QI) initiatives. However, the efficacy of provider incentive programs based on performance has been highly variable.^1^^,^^2^ Attempts to standardize QI efforts around education, audits, and other measures have historically underperformed.^3^^,^^4^ More recently, new modalities of interventions to close clinical care gaps through electronic health records (EHRs) and GDMT-specific programs have garnered significant interest with early promising results or ongoing investigation.^5^^,^^6^

A quality incentive program (QIP) is a pay-for-performance program that incentivizes healthcare providers to meet specific quality metrics based on evidence-based guidelines. These programs may involve financial incentives or nonfinancial incentives, such as professional recognition or leadership roles. With the forthcoming adoption of value-based care models and the proliferation of risk-sharing models from the Centers for Medicare and Medicaid Services and commercial payers, there is an increasing need for physician compensation packages to measure and reimburse for the quality of care provided, rather than the fee-for-service or relative value units metrics currently used in many Academic Medical Centers.^7^

Specialty QIPs have been historically challenging. Potential reasons include difficulty with reliable specialty care patient attribution, as well as challenges in creating accurate disease-specific registries. When implementing a specialty QIP, areas of focus should include prioritizing performance measurement for measures that may yield the greatest opportunities for improvement, offer the highest potential value to clinical outcomes, represent data easily abstracted from the EHR, and align with the goals of both administrative leadership and practicing clinicians.

Given these knowledge gaps in the field, we launched our Cardiovascular Quality Incentive Program (CQIP) with the purpose of improving adherence rates for GDMT for cardiovascular disease (CVD) and heart failure with reduced ejection fraction (HFrEF). We implemented population health tools such as disease-specific dashboards and provider scorecards to improve evidence-based care delivery. We also designed EHR tools to increase the accuracy of identifying patients with specific CVD diagnoses and earn physician trust. GDMT performance metrics were provided to clinicians every quarter through an online dashboard updated monthly, with financial incentives based on their individual performance.

## Methods

### Program design and setting

The design and implementation of the CQIP included a multidisciplinary team of quality leaders, cardiologists, and information technologists. Prior to launch, the group met weekly to design the program and establish the following goals: 1) build and validate an accurate specialty care attribution algorithm; 2) build and validate accurate CVD-specific patient registries; 3) build and validate quality measures in a centralized database; 4) optimize the visualization of the data with an intuitive dashboard and scorecard; 5) create electronic clinical quality measures to mirror performance measures defined in national CVD guidelines^8^, ^9^, ^10^; 6) establish incentive program benchmarks; 7) initiate quarterly reporting to the providers; and 8) disseminate program updates and performance during monthly faculty meetings, lectures, physician onboarding sessions for new hires, and periodic email updates.

The program included 54 noninvasive, interventional, and advanced heart failure cardiologists across 15 ambulatory clinics in an academic health system in Southern California. Based on performance, each clinician was eligible to earn an additional $6,250 quarterly or $25,000 annually (Central Illustration). Baseline performance benchmarks for each measure were established using 24 months of aggregated patient data prior to program launch (July 1, 2019, through June 30, 2021). Cardiologists performing ≥75th percentile received the total eligible amount per measure. Those performing between the 50th and 74th percentiles received half the eligible payment per measure. Those performing <50th percentile but improving at least 6.25% relative to the previous quarter also received half of the eligible amount per measure. Each clinical quality measure was equally weighted for the same amount, and each physician earned a proportional amount per individual metric (eg, an individual achieving 3 out of 6 quality metrics at the 75th percentile or above for a given quarter would earn half the eligible incentive amount). Those not meeting performance described earlier received no incentive payment.

**Central Illustration:**
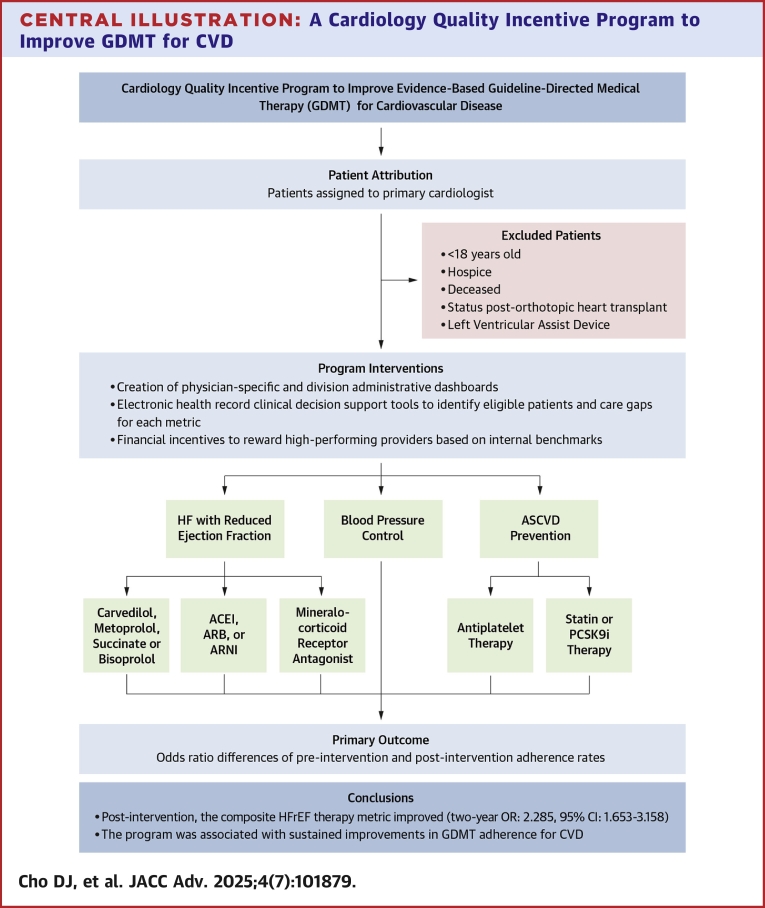
A Cardiology Quality Incentive Program to Improve GDMT for CVD Our Cardiology Quality Incentive Program aimed to improve performance for heart failure with reduced ejection fraction (HFrEF), ASCVD, and blood pressure control. Cardiologists were provided an opportunity to individually earn up to $25,000 annually based on eligible patients’ adherence to a specified beta blocker; ACEI, ARB, or ARNI; and MRA in HFrEF, antiplatelet and statin or PCSK9i therapy for ASCVD risk reduction, and blood pressure control. The study included 54 cardiologists with 75,239 patients over a 4-year period (2 years preintervention, and 2 years postintervention). The composite outcome of HFrEF therapies (2-year OR: 2.285; 95% CI: 1.653-3.158; *P* < 0.001), MRA therapy (2-year OR: 3.039; 95% CI: 2.520-3.663; *P* < 0.001), specified beta blockers (2-year OR: 1.430; 95% CI: 1.129-1.810; *P* = 0.003), ACE, ARB, or ARNI (2-year OR: 1.228; 95% CI: 1.001-1.505; *P* = 0.049), statin or PCSK9i therapies for ASCVD (2-year OR: 1.146; 95% CI: 1.092-1.202; *P* < 0.001), and blood pressure control (2-year OR: 1.496; 95% CI: 1.444-1.550; *P* < 0.001) demonstrated significant and sustained improvement. ASCVD = atherosclerotic cardiovascular disease; CVD = cardiovascular disease; GDMT = guideline-directed medical therapy; MRA = mineralocorticoid receptor antagonist.

### Patient selection and attribution to cardiologist

**Figure 1:**
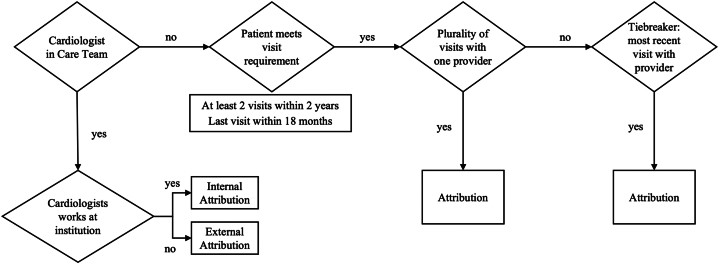
Patient to Cardiologist Attribution Algorithm Attribution algorithm assigning patients to their primary cardiologist. If a patient has seen more than one cardiologist an equal number of times, the algorithm assigns the patient to the provider with the most recent visit.

Empanelment (accurately attributing patients to their unique cardiologist) represented a challenge in a large academic health system, as patients could be seen by multiple specialty physicians. To address this issue, we developed an algorithm to assign each patient to their primary cardiologist (Figure 1). If a cardiologist was assigned to the patient’s care team in the EHR, they were identified as the attributed cardiologist. If no such provider was assigned, we identified patients who had at least 2 office or video visits with the same cardiologist in the last 24 months, with the most recent visit within the preceding 18 months. If a patient had seen multiple providers at least 2 times, the physician with the highest number of ambulatory visits was attributed. In the case of a tie, the physician who saw the patient most recently was attributed. This algorithm was approved by divisional leadership and verified for accuracy prior to program launch by program administrators via thorough chart review, with feedback mechanisms in place if physicians believed they had been incorrectly attributed. When patients were seen by advanced practice providers and cardiovascular fellows, the attending physician on file was still attributed to the patient for the encounter.

### Cardiology quality incentive program

The CQIP dashboard was an online database accessed through an internal website. Providers could visualize their monthly and quarterly performance for each quality measure and compare their individual performance against the entire division. They could access their attributed patient lists for each quality measure, with the default view showing patients with active care gaps. In addition, quarterly scorecards and earned incentive amounts were emailed directly to each provider. Program updates and recognition of the top 10 highest quality performers per quarter were provided at faculty meetings, and provider feedback was solicited regularly.

Quality metrics were categorized into general CVD and HFrEF measures. HFrEF metrics included an active prescription for: 1) specified beta-blockers (carvedilol, bisoprolol, or metoprolol succinate); 2) angiotensin-converting enzyme inhibitor (ACEI), angiotensin receptor blocker (ARB), or angiotensin receptor-neprilysin inhibitor (ARNI); and 3) mineralocorticoid receptor antagonist (MRA). Physician performance for SGLT2 inhibitor (SGLT2i) in HFrEF was monitored but not incorporated into financial incentives for this study. We excluded this metric in our initial program rollout, as our program launched prior to the 2022 American College of Cardiology, American Heart Association, and Heart Failure Society of America guidelines update, which incorporated SGLT2i use in HFrEF as a Class I indication.^11^ We were not confident that improvement in SGLT2i use in HFrEF would be explained solely by our program or be confounded by an increase secondary to updated guidelines.^12^^,^^13^

To increase the capture of the accurate heart failure diagnoses in our registry, an electronic best practice advisory alert was created to assist clinicians in real time. If the patient did not have HFrEF on the problem list but had EHR data suggestive of heart failure, an electronic alert notified the clinician. The alert included an option to select and add the appropriate heart failure diagnosis to the problem list if applicable: HFrEF, heart failure with mildly reduced ejection fraction, or heart failure with preserved ejection fraction. Patients were also categorized as HFrEF if the most recent left ventricular ejection fraction was ≤40%, independent of whether HFrEF was on the problem list.

Atherosclerotic cardiovascular disease (ASCVD) measures included an active prescription for primary and secondary prevention of ASCVD with the use of statin or PCSK9i and secondary ASCVD prevention with use of aspirin or P2Y12 inhibitor. The hypertension metric targeted ambulatory visits with compliance defined as at least one out of the 2 most recent recorded ambulatory office systolic blood pressures <140 mm Hg and diastolic BP <90 mm Hg. While clinical guidelines from professional societies may recommend a lower target of 130 mm Hg for systolic blood pressure and 80 mm Hg for diastolic BP for higher risk patients, we elected to standardize the goal of ≤140/90 mm Hg to align with the Center for Medicare and Medicaid Services Quality Measure ID 236 and University of California Population Health Initiative.^14^^,^^15^

### Inclusion and exclusion criteria

**Figure 2:**
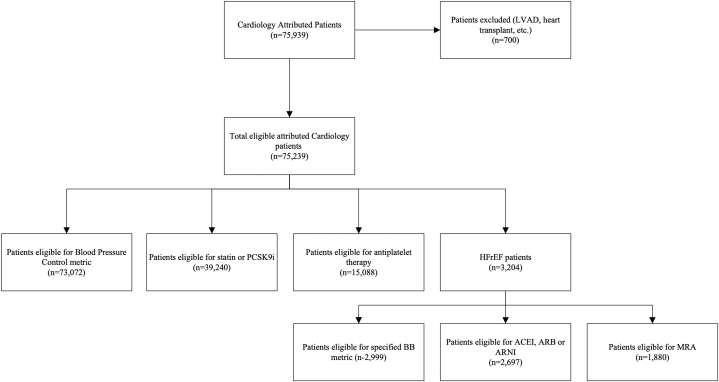
Patient Inclusion and Exclusion for Each CQIP Quality Metric Unique patients attributed to cardiologists eligible for each quality metric in the Cardiovascular Quality Incentive Program (CQIP). BB = beta blocker; HFrEF = heart failure with reduced ejection fraction; LVAD = left ventricular assist device implant; MRA = mineralocorticoid receptor antagonist.

For the HFrEF metric, patients were eligible if they were at least 18 years old and had an HFrEF diagnosis present on their EHR problem list or most recent echocardiogram demonstrating a left ventricular ejection fraction ≤40%. Patients were excluded if they were deceased, on hospice, pregnant, breastfeeding, status post-left ventricular assist device implant, or status post-orthotopic heart transplant. In addition to the aforementioned exclusion criteria, patients with chronic kidney disease stage 4, chronic kidney disease stage 5, end-stage renal disease, or hyperkalemia were also excluded for the ACEI, ARB, ARNI, and MRA metrics (Figure 2).

The antiplatelet metric for secondary ASCVD prevention and statin or PCSK9i use for primary and secondary ASCVD prevention included patients at least 18 years old. Antiplatelet eligibility was determined by the presence of an ASCVD diagnosis on the problem list or past medical history, excluding patients who were deceased, on hospice, already prescribed an anticoagulant, or with a major bleeding diagnosis present on the problem list. Statin or PCSK9i eligibility included patients with: 1) ASCVD diagnosis on the problem list or past medical history; 2) most recent LDL ≥190 mg/dL; 3) patients 40 to 75 years old with diabetes mellitus; or 4) 10-year estimated ASCVD risk score recommending statin initiation. Full descriptions of the inclusion and exclusion criteria utilized for each metric and definitions for HFrEF, heart failure with mildly-reduced ejection fraction, and heart failure with preserved ejection fraction are detailed in Supplemental Tables 1 to 4.

### Study aims

Performance data for the first 24 months of the program (July 1, 2021, through June 30, 2023) was compared against 24 months of baseline performance data preceding program launch. The single exception was the antiplatelet metric for secondary ASCVD prevention, which was limited to 17 months (July 1, 2021 through November 30, 2022) of postintervention data. This was due to an update in the eligible diagnoses for secondary ASCVD, which excluded asymptomatic or nonclinical ASCVD to improve cohort accuracy.

Prescription of GDMT measures that included at least one of the 3 specified beta blockers; ACEI, ARB, or ARNI; or MRA, were aggregated into a composite score defined as HFrEF therapies. We aimed to determine whether our program resulted in a change in performance trends over time for the composite variable and for each individual quality metric. This secondary analysis was formally reviewed by the University of California Los Angeles Institutional Review Board and granted an exemption from institutional review board review per 45 CFR 46.104 (IRB-24-5828).

### Statistical analysis

Descriptive patient statistics are reported in Table 1. To assess change in performance over time, we used an interrupted time series design. We fit mixed-effect binomial regression models with binary variables taking on the value of 1 if the corresponding patient was compliant with a metric, and 0 if they were not, and fixed effects for time, period (preintervention vs postintervention), and time by period interaction. A random effect for provider was included to account for within-provider correlation. Analyses were performed in SAS 9.4, and tables and figures were created in R v4.2.3.

**Table 1:** Patient Descriptive Characteristics

	All Patients (n = 75,239)	Hypertension (n = 56,425)	HFrEF (n = 3,204)	Antiplatelet-Eligible (n = 15,088)	Statin-Eligible (N = 39,240)
Age, y					
Mean (SD)	61.3 (16.3)	64.9 (14.7)	67.2 (15.0)	68.6 (11.7)	67.7 (10.8)
Median (Q1-Q3)	64.0 (51.0-73.0)	67.0 (56.0-75.0)	68.0 (58.0-78.0)	69.0 (62.0-76.0)	68.0 (61.0-74.0)
Min-Max	18.0-104.0	18.0-104.0	18.0-103.0	19.0-103.0	18.0-104.0
Missing	0 (0%)	0 (0%)	0 (0%)	0 (0%)	0 (0%)
Sex					
Male	37,710 (50.1%)	29,632 (52.5%)	2,233 (69.7%)	9,309 (61.7%)	22,575 (57.5%)
Female	37,514 (49.9%)	26,785 (47.5%)	971 (30.3%)	5,779 (38.3%)	16,665 (42.5%)
Missing	15 (0%)	8 (0%)	0 (0%)	0 (0%)	0 (0%)
Race/ethnicity					
Non-Hispanic White	41,663 (55.4%)	30,900 (54.8%)	1,550 (48.4%)	8,843 (58.6%)	22,697 (57.8%)
Non-Hispanic Black	4,303 (5.7%)	3,626 (6.4%)	344 (10.7%)	896 (5.9%)	2,525 (6.4%)
Hispanic	8,859 (11.8%)	6,698 (11.9%)	530 (16.5%)	1,582 (10.5%)	4,186 (10.7%)
Asian	6,919 (9.2%)	5,281 (9.4%)	278 (8.7%)	1,522 (10.1%)	3,624 (9.2%)
Other	2,751 (3.7%)	2099 (3.7%)	158 (4.9%)	522 (3.5%)	1,334 (3.4%)
Missing	10,744 (14.3%)	7,821 (13.9%)	344 (10.7%)	1723 (11.4%)	4,874 (12.4%)
Insurance					
Medicare	29,808 (39.6%)	26,425 (46.8%)	1773 (55.3%)	8,017 (53.1%)	20,333 (51.8%)
Medicaid	2,564 (3.4%)	2034 (3.6%)	317 (9.9%)	348 (2.3%)	1,033 (2.6%)
Private	38,597 (51.3%)	24,842 (44.0%)	965 (30.1%)	5,836 (38.7%)	15,730 (40.1%)
Missing	4,270 (5.7%)	3,124 (5.5%)	149 (4.7%)	887 (5.9%)	2,144 (5.5%)
ASCVD					
No	60,151 (79.9%)	42,836 (75.9%)	2,237 (69.8%)	0 (0.0%)	24,818 (63.2%)
Yes	15,088 (20.1%)	13,589 (24.1%)	967 (30.2%)	15,088 (100.0%)	14,422 (36.8%)
Diabetes					
No	61,969 (82.4%)	43,813 (77.6%)	2,151 (67.1%)	10,684 (70.8%)	29,090 (74.1%)
Yes	13,270 (17.6%)	12,612 (22.4%)	1,053 (32.9%)	4,404 (29.2%)	10,150 (25.9%)
Hypertension					
No	18,814 (25.0%)	0 (0.0%)	21 (0.7%)	1,499 (9.9%)	4,944 (12.6%)
Yes	56,425 (75.0%)	56,425 (100.0%)	3,183 (99.3%)	13,589 (90.1%)	34,296 (87.4%)
Acute myocardial infarction					
No	69,289 (92.1%)	50,714 (89.9%)	2,148 (67.0%)	11,898 (78.9%)	34,282 (87.4%)
Yes	5,950 (7.9%)	5,711 (10.1%)	1,056 (33.0%)	3,190 (21.1%)	4,958 (12.6%)
HFrEF					
No	72,035 (95.7%)	53,242 (94.4%)	0 (0.0%)	14,121 (93.6%)	36,975 (94.2%)
Yes	3,204 (4.3%)	3,183 (5.6%)	3,204 (100.0%)	967 (6.4%)	2,265 (5.8%)
Peripheral vascular disease					
No	71,759 (95.4%)	53,299 (94.5%)	2,991 (93.4%)	13,193 (87.4%)	35,889 (91.5%)
Yes	3,480 (4.6%)	3,126 (5.5%)	213 (6.6%)	1895 (12.6%)	3,351 (8.5%)
CKD stage 4 or above					
No	73,009 (97.0%)	54,210 (96.1%)	2,797 (87.3%)	14,287 (94.7%)	37,685 (96.0%)
Yes	2,230 (3.0%)	2,215 (3.9%)	407 (12.7%)	801 (5.3%)	1,555 (4.0%)
Systolic BP					
Mean (SD)	126.5 (17.6)	129.3 (18.0)	121.6 (20.7)	128.5 (17.8)	129.0 (17.7)
Median (Q1-Q3)	125.0 (114.0-137.0)	128.0 (117.0-140.0)	120.0 (107.0-134.0)	127.0 (116.0-139.0)	128.0 (117.0-140.0)
Min-Max	60.0-246.0	64.0-246.0	65.0-228.0	66.0-231.0	60.0-246.0
Missing	2,745 (3.6%)	2069 (3.7%)	116 (3.6%)	253 (1.7%)	838 (2.1%)
Diastolic BP					
Mean (SD)	74.6 (9.7)	75.0 (10.1)	71.5 (11.3)	73.3 (9.7)	74.5 (9.8)
Median (Q1-Q3)	75.0 (68.0-81.0)	75.0 (69.0-81.0)	71.0 (64.0-78.2)	73.0 (67.0-80.0)	75.0 (68.0-81.0)
Min-Max	6.0-160.0	6.0-160.0	38.0-134.0	30.0-160.0	10.0-160.0
Missing	2,745 (3.6%)	2069 (3.7%)	116 (3.6%)	253 (1.7%)	838 (2.1%)
On hypertensive medication					
No	23,204 (30.8%)	4,390 (7.8%)	44 (1.4%)	2,233 (14.8%)	7,092 (18.1%)
Yes	52,035 (69.2%)	52,035 (92.2%)	3,160 (98.6%)	12,855 (85.2%)	32,148 (81.9%)
Hypertensive med count					
Mean (SD)	1.9 (1.0)	1.9 (1.0)	2.6 (1.1)	2.0 (1.1)	2.0 (1.0)
Median (Q1-Q3)	2.0 (1.0-2.0)	2.0 (1.0-2.0)	3.0 (2.0-3.0)	2.0 (1.0-3.0)	2.0 (1.0-3.0)
Min-Max	1.0-9.0	1.0-9.0	1.0-8.0	1.0-9.0	1.0-9.0
Missing	27,468 (36.5%)	8,654 (15.3%)	117 (3.7%)	3,277 (21.7%)	9,563 (24.4%)
Aspirin or P2Y12 inhibitor					
No	63,375 (84.2%)	45,421 (80.5%)	2,352 (73.4%)	3,224 (21.4%)	27,870 (71.0%)
Yes	11,864 (15.8%)	11,004 (19.5%)	852 (26.6%)	11,864 (78.6%)	11,370 (29.0%)
Statin or PCSK9i					
No	42,953 (57.1%)	27,516 (48.8%)	1,198 (37.4%)	1749 (11.6%)	6,954 (17.7%)
Yes	32,286 (42.9%)	28,909 (51.2%)	2006 (62.6%)	13,339 (88.4%)	32,286 (82.3%)
Specified beta blocker					
No	72,427 (96.3%)	53,613 (95.0%)	392 (12.2%)	14,232 (94.3%)	37,232 (94.9%)
Yes	2,812 (3.7%)	2,812 (5.0%)	2,812 (87.8%)	856 (5.7%)	2008 (5.1%)
ACEI, ARB, or ARNI					
No	72,830 (96.8%)	54,021 (95.7%)	795 (24.8%)	14,361 (95.2%)	37,499 (95.6%)
Yes	2,409 (3.2%)	2,404 (4.3%)	2,409 (75.2%)	727 (4.8%)	1741 (4.4%)
MRA					
No	73,926 (98.3%)	55,112 (97.7%)	1891 (59.0%)	14,741 (97.7%)	38,323 (97.7%)
Yes	1,313 (1.7%)	1,313 (2.3%)	1,313 (41.0%)	347 (2.3%)	917 (2.3%)
SGLT2i					
No	74,133 (98.5%)	55,320 (98.0%)	2098 (65.5%)	14,754 (97.8%)	38,410 (97.9%)
Yes	1,106 (1.5%)	1,105 (2.0%)	1,106 (34.5%)	334 (2.2%)	830 (2.1%)

ASCVD = atherosclerotic cardiovascular disease; BP = blood pressure; CKD = chronic kidney disease; HFrEF = heart failure with reduced ejection fraction; MRA = mineralocorticoid receptor antagonist; PCSK9i = proprotein convertase subtilisin/kexin type 9 inhibitor; SGLT2i = SGLT2 inhibitor.

## Results

Fifty-four noninvasive, interventional, and advanced heart failure cardiologists were included in the CQIP across 15 ambulatory clinics over a 24-month period after the program launch. A total of 75,239 patients with a mean age of 61.3 ± 16.3 years were included, of which 37,710 patients (50.1%) were male (Table 1). Comorbidities included 56,425 (75%) patients with diagnosed hypertension, 13,270 (17.6%) patients with diabetes mellitus, 15,088 (20.1%) patients with ASCVD diagnoses, 5,950 (7.9%) patients with a history of myocardial infarction, 3,204 (4.3%) patients with HFrEF, and 39,240 (52.2%) patients eligible for statin or PCSK9i therapy.

Race and ethnicity included 41,663 (55.4%) non-Hispanic White patients, 4,303 (5.7%) non-Hispanic Black patients, 8,859 (11.8%) Hispanic patients, 6,919 (9.2%) Asian patients, 2,751 (3.7%) other patients, while 10,744 (14.3%) patients had no race or ethnicity on file.

Table 2 describes the adherence rates and odds ratios (ORs) from fitted models. The preintervention and postintervention rates for each start and end period (preintervention July 2019 and June 2021) contain adherence rates and monthly trend differences as estimated from interrupted time series models. Monthly ORs can be interpreted as the change in odds of adherence from 1 month to the next (ie, a monthly preintervention OR of 1.01 indicates a 1% increase in the odds of adherence per month during the preintervention period). The difference columns contain the per-month, per-1-year, and per-2-year multiplicative change in the odds when comparing the postintervention period to the preintervention period. For example, a value of 1.01 in the monthly difference column indicates that the odds of adherence increase 1% more per month in the postintervention period than in the preintervention period.

**Table 2:** Adherence Rates and ORs From Fitted Models

Measure	Preintervention Period (July 1, 2019-June 30, 2021)			Postintervention Period (July 1, 2021-June 30, 2023)		
	Start Adherence Rate	Monthly OR (95% CI)	End Adherence Rate	Start Adherence Rate	Monthly OR (95% CI)	End Adherence Rate
Composite GDMT for HFrEF (at least one)	98.4%	0.992 (0.983-1.002)	98.1%	98.1%	1.027 (1.017-1.037)	99.0%
Statin or PCSK9i	77.6%	1.006 (1.005-1.008)	79.8%	79.8%	1.012 (1.011-1.013)	83.9%
Aspirin or P2Y12 inhibitor	78.8%	1.004 (1.001-1.007)	79.9%	79.0%	0.993 (0.990-0.995)	77.0%
Specified beta blocker	93.3%	1.007 (1.000-1.014)	94.2%	94.1%	1.022 (1.015-1.029)	96.3%
ACE, ARB, or ARNI	88.9%	1.008 (1.002-1.014)	90.6%	90.9%	1.017 (1.011-1.023)	93.6%
MRA	55.9%	0.996 (0.991-1.002)	53.7%	59.4%	1.043 (1.038-1.049)	79.5%
Blood pressure control	89.0%	0.991 (0.990-0.992)	86.9%	87.4%	1.008 (1.007-1.009)	89.3%

Measure	OR Difference Between Preintervention and Postintervention Periods			
	Monthly Post vs Pre OR (95% CI)	Yearly Post vs Pre OR (95% CI)	2-Year Post vs Pre OR (95% CI)	*P* Value
Composite GDMT for heart failure (at least one)	1.035 (1.021-1.049)	1.512 (1.286-1.777)	2.285 (1.653-3.158)	<0.001
Statin or PCSK9i	1.006 (1.004-1.008)	1.070 (1.045-1.096)	1.146 (1.092-1.202)	<0.001
Aspirin or P2Y12 inhibitor	0.988 (0.985-0.992)	0.870 (0.830-0.911)	0.757 (0.689-0.831)	<0.001
Specified beta blocker	1.015 (1.005-1.025)	1.196 (1.063-1.345)	1.430 (1.129-1.810)	0.003
ACE, ARB, or ARNI	1.009 (1.000-1.017)	1.108 (1.001-1.227)	1.228 (1.001-1.505)	0.049
MRA	1.047 (1.039-1.056)	1.743 (1.588-1.914)	3.039 (2.520-3.663)	<0.001
Blood pressure control	1.017 (1.015-1.018)	1.223 (1.202-1.245)	1.496 (1.444-1.550)	<0.001

The preintervention and postintervention columns contain adherence rates and monthly trend differences as estimated from interrupted time series models. Monthly ORs here can be interpreted as the change in odds of adherence from 1 month to the next, for example, a monthly preintervention OR of 1.01 indicates a "1% increase in the odds of adherence per month during the preintervention period." The difference columns contain the difference in trends expressed in monthly, yearly, and 2-year ORs. A monthly difference OR of 1.04 here for example can be interpreted as a "4% increase in the monthly odds of adherence from preintervention to postintervention," and a yearly difference OR of 1.50 can be interpreted as a “50% increase in the yearly odds of adherence from preintervention to postintervention.”

GDMT = guideline-directed medical therapy; HFrEF = heart failure with reduced ejection fraction; MRA = mineralocorticoid receptor antagonist.

**Figure 3:**
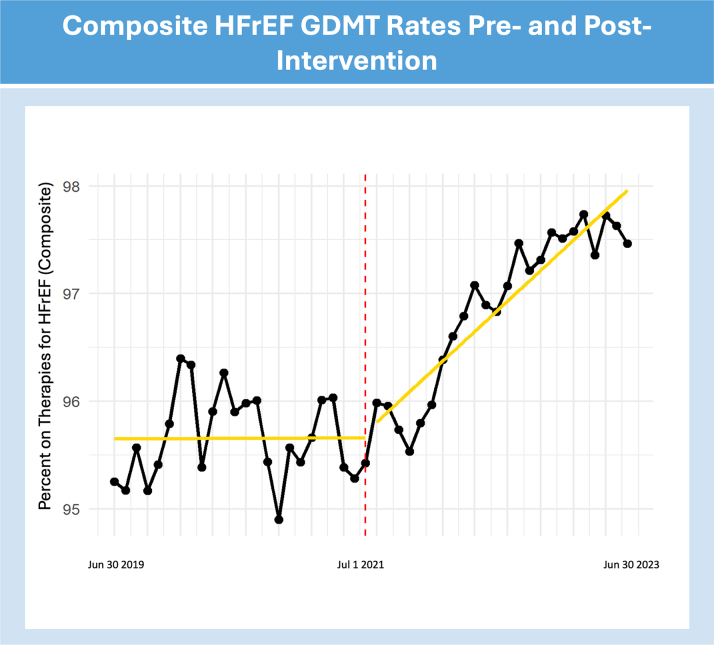
Impact of a CQIP on Composite GDMT for HFrEF Preintervention and postintervention adherence rates for the composite outcome of GDMT in HFrEF, indicated by the presence of at least one of the 3 GDMT treatments (specified beta blocker, ACEI, ARB or ANI and MRA). The vertical red line marks the start date of the CQIP. CQIP = Cardiovascular Quality Incentive Program; GDMT = guideline-directed medical therapy; HFrEF = heart failure with reduced ejection fraction; MRA = mineralocorticoid receptor antagonist.

The OR difference for HFrEF composite therapies (Figure 3) demonstrated a per-month OR of 1.035 (95% CI: 1.021-1.049; *P* < 0.001), per-1-year OR 1.512 (95% CI: 1.286-1.777; *P* < 0.001), and per-2-year OR 2.285 (95% CI: 1.653-3.158; *P* < 0.001). This may be interpreted as a 3.5% increase in the monthly odds of adherence, 51.2% increase in the per-1-year odds of adherence, and 128.5% increase in the per-2-year odds of adherence from preintervention to postintervention.

**Figure 4:**
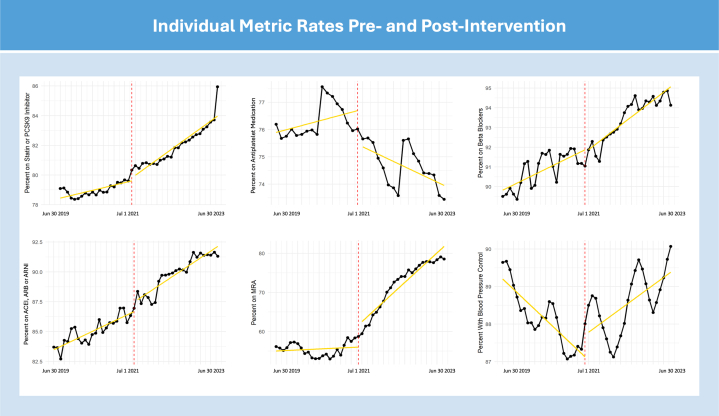
Impact of CQIP on Individual Quality Metrics for HFrEF, ASCVD, and Blood Pressure Control Pre-intervention and post-intervention adherence rates for the individual quality metrics for blood pressure control, antiplatelet and statin or PCSK9i therapy for ASCVD, and specified beta blocker; ACEI, ARB, or ARNI; and MRI for HFrEF. The vertical red line marks the start date of the CQIP. ASCVD = atherosclerotic cardiovascular disease; CQIP = Cardiovascular Quality Incentive Program; HFrEF = heart failure with reduced ejection fraction; MRA = mineralocorticoid receptor antagonist.

For individual metrics (Figure 4), the OR difference was most notable for MRA use, demonstrating a per-month OR of 1.047 (95% CI: 1.039-1.056; *P* < 0.001), per-1-year OR 1.743 (95% CI: 1.588-1.1914; *P* < 0.001), and a per-2-year OR 3.039 (95% CI: 2.520-3.663; *P* < 0.001). Specified beta blockers demonstrated a per-month OR of 1.015 (95% CI: 1.005-1.025; *P* = 0.003), per-1-year OR 1.196 (95% CI: 1.063-1.345; *P* = 0.003), and a per-2-year OR 1.430 (95% CI: 1.129-1.810; *P* = 0.003). ACE, ARB, or ARNI demonstrated a per-month OR of 1.009 (1.000-1.017; *P* = 0.049), per-1-year OR 1.108 (95% CI: 1.001-1.227; *P* = 0.049), and a per-2-year OR 1.228 (95% CI: 1.001-1.505; *P* = 0.049).

Statin or PCSK9i use demonstrated improved odds ratio differences over time as well, with a per-month OR of 1.006 (95% CI: 1.004-1.008; *P* < 0.001), per-1-year OR 1.070 (95% CI: 1.045-1.096; *P* < 0.001) and per-2-year OR 1.146 (95% CI: 1.092-1.202; *P* < 0.001). Blood pressure control showed an improving OR difference as well, with a per-month OR of 1.017 (95% CI: 1.015-1.018; *P* < 0.001), per-year OR of 1.223 (95% CI: 1.202-1.245; *P* < 0.001), and 2-year OR of 1.496 (95% CI: 1.444-1.550; *P* < 0.001). Of note, the antiplatelet metric noted a decreasing OR difference between the preintervention and postintervention periods, with a per-2-year OR of 0.757 (95% CI: 0.689-0.831; *P* < 0.001).

## Discussion

Population health tools that are designed and implemented into clinical practice, such as clinical dashboards, provider scorecards, and EHR tools, allow for the ability to identify and close clinical care gaps at both the population and individual patient levels. Our CQIP provided clinicians with tools to evaluate and compare their performance relative to the entire division and provided the ability to identify and close care gaps retrospectively and prospectively. Physicians received objective feedback on the quality of care they provided, with mechanisms to provide continuous feedback and improvement to the program leaders. We found that combining our database and EHR tools with financial incentives was associated with an improvement in adherence to GDMT.

Historically, QI interventions to improve adherence to GDMT for CVD have not consistently shown benefit. In a prior systematic review, audit and feedback and educational outreach mechanisms were generally effective for process measures (ie, prescription of medication) and clinical effectiveness measures (ie, hard clinical outcomes), but clinical reminders and provider incentives had mixed results.^16^ For heart failure specifically, remote GDMT optimization programs, patient prompts, and provider-level EHR nudges improved process measures,^5^^,^^17^^,^^18^ while hospital-based feedback and education programs did not lead to improved heart failure quality metrics or clinical outcomes.^3^^,^^4^^,^^19^ For adherence to cholesterol guidelines, studies have had heterogenous results; however, interventions based on physician education with clinical decision support systems or audit/feedback systems, as well as those based on patient education alone, have led to improved statin prescription rates.^20^, ^21^, ^22^ Quality-improvement programs have targeted blood pressure control with positive improvements in the patients reaching their hypertension goals, including programs utilizing a team-based approach with pharmacist-led programs; however, only 1 in 4 adults in the United States have controlled hypertension.^18^^,^^23^^,^^24^ Financial incentives for primary CVD prevention have shown improved blood pressure control, but their impact on other CVD metrics is unknown.^25^

To our knowledge, this is the first study evaluating the effectiveness of leveraging EHR population health tools with financial incentives to improve and sustain performance on CVD quality metrics. Over 24 months, we found that overall composite GDMT therapies, most notably MRA adherence, in addition to specified beta blocker use; ACE, ARB, and ARNi adherence; statin or PCSK9i adherence, and blood pressure control improved after program initiation with a durable effect.

We hypothesize that a vital component to the success of our program was engaging academic cardiologists in the study by putting together accurate patient-level data and working with physicians to build trust and collaboration in evaluation of their performance with GDMT. Prior to the CQIP and development of our cardiology dashboard, providers lacked the ability to monitor their individual performance for CVD quality measures and received no financial incentive or recognition for providing high-quality care. To ensure our program quickly gained the buy-in of participating cardiologists, we created an accurate patient attribution algorithm, optimized patient registries using novel EHR tools, and thoroughly validated each performance metric with designated group of physicians, including cardiologists. The accuracy of the patient cohorts and measures, the transparency of comparator quality data to cardiologist end-users, and financial incentives to reward high-quality care were all critical to the program’s overall success. In addition, our program provided clinicians with their first mechanism to readily identify patients on their panels with active clinical care gaps retrospectively and at the point of care, allowing for immediate care gap closure or the necessary follow-up to address GDMT gaps. When frontline cardiologists were prompted to add medications to patient regimens, they reported high confidence in the appropriateness of the recommendations.

We also ensured that changes were implemented without creating additional administrative burden. We realized that cardiologists were the stakeholders with the ability to close clinical care gaps, but that they also had the least amount of time available to do so. By pairing financial incentives with time-saving tools and actionable data, we created a culture that allowed physicians to claim ownership of their panel of patients and augment their yearly compensation by being held accountable to universally accepted CVD quality metrics. As our outcomes evaluated the effect of our program over 24 months, our results suggest that the positive trend in GDMT treatment is sustained over time, with mean rates that initially improved and continued to improve over our study period. By actively refining the implementation process and the incentive mechanisms, we believe that sustained success in driving adherence to GDMT and other important cardiology metrics is achievable.

The increase in our composite score for GDMT was mainly driven by an increased prescription rate of MRAs, which historically have been underutilized in HFrEF patients and were a notable target for improved GDMT in similar studies.^26^ In addition, our baseline rates for both specified beta blockers and ACEI, ARB, and ARNI metrics started at high rates of adherence prior to program initiation, suggesting that the magnitude of effect would be smaller than that of MRA improvement. Regarding ASCVD prevention, we noticed a sustained improvement in patients prescribed a statin or PCSK9i for primary and secondary prevention, while our antiplatelet metric demonstrated decreasing trends after program initiation. We theorize that the change in practice patterns and guidelines limiting aspirin for true secondary ASCVD diagnoses and high-risk ASCVD patients led to this decline over time. As noted earlier, our study period for the intervention for aspirin was limited to 17 months, as we upgraded our program cohort for the antiplatelet metric to a more limited set of diagnoses and criteria for secondary ASCVD prevention to maintain high accuracy of eligible patients.

We recognize that patient populations with CVD are dynamic and changing over time, with new patients entering the clinical care pathways and others phasing out or continuing within the population. To account for potential changes in patient demographics over time and the potential impact on our time-series analysis, we evaluated patient demographics in the preintervention and postintervention periods (Table 3). We noted that both patient groups are comparable in terms of their characteristics, making it unlikely that changing population dynamics over time may have affected our results.

**Table 3:** Patient Demographics Preintervention and Postintervention

	Pre (n = 46,570)	Post (n = 61,821)	Standardized Mean Difference
Age, y			0.014
Mean (SD)	62.2 (16.1)	62.4 (16.2)	
Median (Q1-Q3)	64.0 (52.0-74.0)	65.0 (53.0-74.0)	
Min-Max	18.0-104.0	18.0-106.0	
Missing	0 (0%)	0 (0%)	
Sex			0.015
Male	23,788 (51.1%)	31,093 (50.3%)	
Female	22,782 (48.9%)	30,713 (49.7%)	
Missing	0 (0%)	15 (0%)	
Race/ethnicity			0.07
Non-Hispanic White	26,287 (56.4%)	34,286 (55.5%)	
Non-Hispanic Black	2,769 (5.9%)	3,474 (5.6%)	
Hispanic	5,357 (11.5%)	7,283 (11.8%)	
Asian	4,261 (9.1%)	5,682 (9.2%)	
Other	2032 (4.4%)	1912 (3.1%)	
Missing	5,864 (12.6%)	9,184 (14.9%)	
Insurance			0.061
Medicare	19,069 (40.9%)	26,399 (42.7%)	
Medicaid	1,189 (2.6%)	2,263 (3.7%)	
Private	22,868 (49.1%)	30,663 (49.6%)	
Missing	3,444 (7.4%)	2,496 (4%)	
Systolic BP			0.046
Mean (SD)	126.3 (17.5)	127.1 (17.6)	
Median (Q1-Q3)	125.0 (114.0-137.0)	126.0 (115.0-137.0)	
Min-Max	60.0-246.0	60.0-225.0	
Missing	840 (1.8%)	2,640 (4.3%)	
Diastolic BP			0.044
Mean (SD)	74.3 (9.8)	74.8 (9.7)	
Median (Q1-Q3)	74.0 (68.0-81.0)	75.0 (69.0-81.0)	
Min-Max	6.0-141.0	0.0-160.0	
Missing	840 (1.8%)	2,640 (4.3%)	
Hypertensive med count			0.039
Mean (SD)	1.9 (1.0)	1.9 (1.0)	
Median (Q1-Q3)	2.0 (1.0-2.0)	2.0 (1.0-2.0)	
Min-Max	1.0-9.0	1.0-8.0	
Missing	15,934 (34.2%)	21,333 (34.5%)	
ASCVD			0.008
No	36,914 (79.3%)	48,790 (78.9%)	
Yes	9,656 (20.7%)	13,031 (21.1%)	
Diabetes			0.006
No	38,329 (82.3%)	50,751 (82.1%)	
Yes	8,241 (17.7%)	11,070 (17.9%)	
Hypertension			0.005
No	11,266 (24.2%)	14,822 (24.0%)	
Yes	35,304 (75.8%)	46,999 (76.0%)	
Acute myocardial infarction			0.035
No	42,464 (91.2%)	56,975 (92.2%)	
Yes	4,106 (8.8%)	4,846 (7.8%)	
HFrEF			0.011
No	44,759 (96.1%)	59,278 (95.9%)	
Yes	1811 (3.9%)	2,543 (4.1%)	
Peripheral vascular disease			0.082
No	44,990 (96.6%)	58,711 (95.0%)	
Yes	1,580 (3.4%)	3,110 (5.0%)	
CKD stage 4 or above			0.046
No	45,057 (96.8%)	60,288 (97.5%)	
Yes	1,513 (3.2%)	1,533 (2.5%)	
On hypertensive med			0.001
No	14,073 (30.2%)	18,714 (30.3%)	
Yes	32,497 (69.8%)	43,107 (69.7%)	
Aspirin or P2Y12 inhibitor			0.001
No	38,941 (83.6%)	51,721 (83.7%)	
Yes	7,629 (16.4%)	10,100 (16.3%)	
Statin or PCSK9i			0.166
No	28,816 (61.9%)	33,189 (53.7%)	
Yes	17,754 (38.1%)	28,632 (46.3%)	
Specified beta blocker			0.014
No	45,015 (96.7%)	59,596 (96.4%)	
Yes	1,555 (3.3%)	2,225 (3.6%)	
ACEI, ARB, or ARNI			0.017
No	45,255 (97.2%)	59,901 (96.9%)	
Yes	1,315 (2.8%)	1920 (3.1%)	
MRA			0.035
No	45,960 (98.7%)	60,750 (98.3%)	
Yes	610 (1.3%)	1,071 (1.7%)	
SGLT2i			0.101
No	46,281 (99.4%)	60,768 (98.3%)	
Yes	289 (0.6%)	1,053 (1.7%)	

ASCVD = atherosclerotic cardiovascular disease; BP = blood pressure; CKD = chronic kidney disease; HFrEF = heart failure with reduced ejection fraction; MRA = mineralocorticoid receptor antagonist; SGLT2i = SGLT2 inhibitor.

In addition, we considered that treatment-naive patients may receive more intense treatment than existing patients, providing fewer opportunities for providers with mature panels to improve care metrics. However, providers are not incentivized by rate of care gap closure, but by maintaining a high percentage (>75th percentile to receive full eligible amount per metric), although we realize that the rate of improvement over time may continue to slow if all providers achieved high performance.

As our program continues to evolve, we specifically designed the underlying infrastructure to be adaptable for future updates to clinical guidelines and continued improvement from further quality-improvement plan-do-study-act cycles that incorporate more efficient clinical workflows and emerging technologies.

### Study Limitations

This study relied on 2 factors that were vital to its success: the rapid and accurate production of population health tools based on the EHR and financial incentives for performance on quality measures. The generalizability of this study to other healthcare environments depends on institutional strategic vision paired with clinician and IT support to build, maintain, and continuously improve program components. The individual contribution of financial incentives alone compared to the creation of the data infrastructure for population health management of CVD for the overall and sustained improvement of the CQIP is difficult to measure, and both are likely critical for success at our institution. In addition, our program targeted equal opportunities for improvement among all physicians, and we did not evaluate for variations in rates of improvement among noninvasive, interventional, and advanced heart failure cardiologists, nor did we evaluate variations in rates by geography or clinical productivity, although these areas are currently being studied. We did not evaluate whether targeted interventions towards the lowest performers would have shown a larger magnitude of improvement. Formal evaluation of provider feedback preintervention and postintervention was not conducted, although program leadership did solicit regular input for improvement. Evaluation of disparities among patients was not within the scope of this study period but is an area for further study and under active investigation.

Our study did not distinguish between patients who were newly referred to a cardiologist and those who were already established, limiting our ability to assess whether treatment-naive patients—particularly those with HFrEF—were more likely to receive interventions targeting care gap closures than existing patients, who may have had fewer opportunities for treatment intensification. To explore whether differences in patient panel composition could have influenced our findings, we conducted additional mixed-effects models for each of the 7 outcomes, incorporating a fixed effect for the three-way interaction between time, intervention period (pre- vs post-), and clinician career stage (defined as <3 years vs ≥3 years in practice). This approach allowed us to test whether the observed effects varied according to clinician experience, which may be a proxy for the maturity of their patient panel. After applying Bonferroni correction for multiple comparisons, none of the three-way interaction terms reached statistical significance (smallest adjusted *P* value 7 × 0.02 = 0.14). These findings suggest that clinician career stage—and, by extension, patient panel composition—did not meaningfully influence the observed impact of the intervention.

Although our primary mixed-effects models accounted for provider-level variability through random effects, we did not explicitly model autocorrelation. We selected piecewise linear models over Autoregressive Integrated Moving Average models to enhance interpretability for clinicians and health system stakeholders. To assess the robustness of our findings, we conducted a sensitivity analysis using generalized estimating equations (GEEs), clustering at the provider level and assuming an independent correlation structure. For inference, we used cluster-robust standard errors, which offer a more conservative approach and are robust to both autocorrelation and within-provider correlation. Results from the GEE models, presented alongside our primary mixed-effects models in Supplemental Table 6, showed comparable effect sizes. While a few effects that were statistically significant in the mixed-effects models were no longer statistically significant in the GEE models—likely due to the reduced efficiency of cluster-robust standard errors—the overall pattern of findings remained consistent. These results suggest that our primary conclusions are not driven by model assumptions about correlation structure, but rather reflect the true impact of the incentive program to improve quality measures for CVD.

The prescription dose of GDMT medications in HFrEF or the addition or uptitration of antihypertensive medications was not studied. Barriers to patient access for PCSK9i, such as cost and insurance approval, were not measured or evaluated in this study. Importantly, there was no randomized concurrent control group, so secular trends in use or other factors could have impacted some or all the findings. A future study that includes such a control group would address the issue of time-varying confounding that can influence the results of the interrupted time series models we implemented in this study. However, to our knowledge, our CQIP was the only initiative in our health system during the study period likely to affect the adherence metrics analyzed. This strengthens our confidence that the trends observed prior to the intervention provide a reasonable basis for comparison with the postintervention period. To further reduce the risk of confounding, we carefully selected metrics that were unlikely to be influenced by external changes. As described earlier, although physician performance on SGLT2i use in HFrEF was measured, we excluded this metric from the incentive program and analysis of this study due to updated American College of Cardiology/American Heart Association/Heart Failure Society of America guidelines issued shortly after program launch, which introduced a Class I recommendation for SGLT2i use. This exclusion helped ensure that improvements observed in our study were more likely attributable to the intervention itself.

Other guideline-based recommendations for patients with CVD, including pneumococcal and influenza vaccinations, have not been implemented for financial incentives but are currently being measured for future potential interventions. In addition, our hypertension metric relied on ambulatory blood pressure measurements, which are less accurate than longitudinal home blood pressure measurements over time.^27^ Incorporation of digital health technologies such as artificial intelligence and remote monitoring technologies for improved cohort identification, adherence, and uptitration of therapies for CVD and HFrEF is under future consideration and evaluation for continued improvement in our CQIP.

## Conclusions

The implementation of a CQIP utilizing monetary and nonfinancial incentives, coupled with transparent and easily accessible data visualization, was associated with notable improvements in GDMT measures for CVD. The incorporation of the cardiology dashboard and scorecard, based on accurate and validated performance metrics with EHR tools, likely played a critical role in enabling physicians to confidently identify and close clinical care caps. The insights garnered from our study may serve as a foundation for other healthcare systems aiming to bolster GDMT adherence through QIPs.

Perspectives **COMPETENCY IN PATIENT CARE, SYSTEMS-BASED PRACTICE, LEADERSHIP, AND ADMINISTRATIVE COMPETENCIES:** A cardiology quality-improvement program supported by financial incentives and built upon a supportive and accurate IT infrastructure can be successfully designed and implemented to improve and sustain GDMT performance for CVD and HFrEF. Our program helped clinicians monitor and improve their individual performance while identifying areas of improvement, and it may serve as a model for the implementation of ambulatory cardiovascular quality programs on a broader scale.

**TRANSLATIONAL OUTLOOK:** Further studies are needed to validate the feasibility and reproducibility of a financially incentivized quality program, reinforced by robust IT infrastructure, to drive meaningful improvements in GDMT for CVD.

## Funding support and author disclosures

The project described was supported within the institution by the 10.13039/100019688Department of Medicine at 10.13039/100008340UCLA Health to provide the financial incentive payments for each eligible provider in the quality incentive program, information technology analyst support and statistical support for data analysis. Dr Fonarow has consulted for 10.13039/100000046Abbott, 10.13039/100002429Amgen, 10.13039/100004325AstraZeneca, Bayer, 10.13039/100001003Boehringer Ingelheim, 10.13039/100014941Cytokinetics, Eli Lilly, Johnson & Johnson, 10.13039/100004374Medtronic, 10.13039/100004334Merck, Novartis, and Pfizer. Dr Hsue received honoraria from Genentech, Pfizer, Gilead, and Merck unrelated to the topic of this study; study drug provided by Eli Lilly. Dr Chima-Melton has consulted for AstraZeneca, Boehringer Ingelheim, and Gilead. All other authors have reported that they have no relationships relevant to the contents of this paper to disclose.
