# Supplementary material for: Implementing a Cardiology Quality Incentive Program to Improve Guideline-Directed Medical Therapy
Source: JACC Adv. 2025 Jun 20;4(7):101879. doi: 10.1016/j.jacadv.2025.101879 (PMC12221717; doi:10.1016/j.jacadv.2025.101879)
Supplement: Supplemental Material [file mmc1.docx]

Supplemental Table 1: Quality Metrics Definitions with Inclusion Criteria, Exclusion Criteria, and Adherence Algorithm

| **Measure Name** | **Description** | **Denominator Exclusions** |
| --- | --- | --- |
| Statin or PCSK9 inhibitor for primary and secondary ASCVD prevention | Among cardiology attributed patients with statin indication in health maintenance, the percent of patients on statin or PCSK9 inhibitor (evolocumab, alirocumab or inclisiran). | - Pregnant Patients - Breastfeeding patients - Hospice Patients - VAD Patients - Heart Transplant Patients - Statin or PCSK9 inhibitor allergy |
| Antiplatelet therapy for secondary ASCVD prevention | Among cardiology attributed patients with aspirin indication in health maintenance, the percent on antiplatelet therapy (aspirin, P2Y12 inhibitor or dipyridamole) as indicated in Health maintenance. | - Pregnant Patients - Breastfeeding patients - Hospice Patients - VAD Patients - Heart Transplant Patients - Patients with High-Risk Bleeding Diagnoses on Problem List - Currently on Anticoagulation Medication |
| Blood Pressure Control | Among cardiology attributed patients, aged 18 and above and two BP readings in the past 3 years, the percent with the last ambulatory BP readings less than 140/90 within the last 3 years.   - For 2+ BPs in one day, select BP with lowest MAP - For numerator compliance, there must be two consecutive office BP readings where at least 1 reading is less than 140/90 on separate days. - For numerator non-compliance, there must be two consecutive office BP readings greater than 140/90 on separate days. | - Hospice Patients - VAD Patients - Heart Transplant Patients - White Coat Hypertension Diagnosis on Problem List |
| HFrEF: Carvedilol, Metoprolol Succinate or Bisoprolol | Among cardiology attributed patients with active HFrEF Diagnosis in the problem list or most recent LVEF≤40%, the percentage of patients on specific HFrEF indicated beta blockers.  In denominator eligible patients, use the algorithm below:   - If HFrEF present on problem list (regardless of LVEF %) AND current meds includes carvedilol, metoprolol succinate or bisoprolol, include in the denominator. This will be a numerator compliance to account for HF with improved EF patients. - If most recent LVEF≤40% AND current meds do not include specified beta blockers, include in the denominator. This will be a numerator non-compliance. | - Pregnant Patients - Breastfeeding patients - Hospice Patients - VAD Patients - Heart Transplant Patients - Patients with HFrEF present on problem list AND current meds do not include specified BB AND patient does not have an LVEF on file OR most recent LVEF>40% |
| HFrEF: ACEI, ARB or ARNI | Among cardiology attributed patients with active HFrEF Diagnosis in the problem list or most recent LVEF≤40%, the percentage of patients on ACE or ARB or ARNI.  In presenting denominator eligible patients, use the algorithm below:   - If HFrEF present on problem list (regardless of LVEF%) AND current meds includes ACE or ARB or ARNI, include in the denominator. This will be a numerator compliance to account for HF with improved EF patients. - If most recent LVEF≤40% AND current meds do not include ACEI, ARB or ARNI, include in the denominator. This will be a numerator non-compliance. | - Hyperkalemia - CKD Stage 4 - CKD Stage 5 - CKD ESRD - Pregnant Patients - Breastfeeding patients - Hospice Patients - VAD Patients - Heart Transplant Patients - Patients with HFrEF present on problem list AND current meds do not include ACE, ARB or ARNI AND patient does not have an LVEF on file OR LVEF>40% |
| HFrEF: MRA | Among cardiology attributed patients with active HFrEF Diagnosis in the problem list or most recent LVEF≤40%, the percentage of patients on MRA.  In presenting denominator eligible patients, use the algorithm below:   - If HFrEF present on problem list (regardless of LVEF%) AND current meds includes MRA (spironolactone or eplerenone), include in the denominator. This will be a numerator compliance to account for HF with improved EF patients. - If most recent LVEF≤35% AND current meds DO NOT include MRA (spironolactone or eplerenone), include in the denominator. This will be a numerator non-compliance | - Hyperkalemia - CKD Stage 4 - CKD Stage 5 - CKD ESRD - Pregnant Patients - Breastfeeding patients - Hospice Patients - VAD Patients - Heart Transplant Patients - Patients with HFrEF present on problem list AND current meds do not include MRA patient does not have an LVEF on file OR LVEF>35% |
| HFrEF: SGLT2I | Among cardiology attributed patients with active HFrEF Diagnosis in the problem list or most recent LVEF≤40%, the percentage of patients on SGLT2I.  In presenting denominator eligible patients, use the algorithm below:   - If HFrEF present on problem list (regardless of LVEF%) AND current meds includes SGLT2I, include in the denominator. This will be a numerator compliance to account for HF with improved EF patients. - If most recent LVEF≤40% AND current meds do not include SGLT2I, include in the denominator. This will be a numerator non-compliance. | - Hospice Patients - Heart Transplant Patients - VAD Patients - Pregnant Patients - Breastfeeding Patients - Diabetes Type 1 - CKD Stage 4 - CKD Stage 5 - CKD ESRD - Patients with HFrEF present on problem list AND current meds do not include SGLT2I AND patient does not have an LVEF on file OR LVEF>40% |

Supplemental Table 2: Heart Failure with Reduced Ejection Fraction Diagnoses

| **Diagnosis Code Type** | **Diagnosis Code** | **Example Diagnosis Description** |
| --- | --- | --- |
| ICD-10 | I11.0 | Systolic heart failure secondary to hypertension |
|  | I13.X | Hypertensive heart and kidney disease with systolic CHF, NYHA class 3 and ESRD |
|  | I25.10 | Systolic heart failure secondary to coronary artery disease |
|  | I38 | Chronic systolic heart failure due to valvular disease |
|  | I42.X | Systolic heart failure secondary to hypertrophic obstructive cardiomyopathy |
|  | I50.1 | Left heart failure with left ejection fraction less than or equal to 30 percent |
|  | I50.2X | Clinical systolic heart failure |
|  | I50.4X | Combined systolic and diastolic congestive heart failure |
|  | I50.814 | Biventricular heart failure with reduced left ventricular function |
|  | I50.9 | Heart failure with reduced left ventricular function, NYHA class 2 |
|  | N18.X | Benign hypertensive heart and kidney disease with systolic CHF, NYHA class 2 and CKD stage 1 |
|  | Z99.2 | Hypertensive heart disease with combined systolic and diastolic heart failure and end stage chronic kidney disease on dialysis |
| ICD-9 | 401 | Malignant essential hypertension with congestive heart failure with combined systolic and diastolic dysfunction (HCC/RAF) |
|  | 402.X | Accelerated hypertension with NYHA class 4 systolic heart failure (HCC/RAF) |
|  | 403 | Malignant systolic hypertension with CHF and chronic kidney disease (HCC/RAF) |
|  | 404.X | Malignant hypertensive heart and kidney disease with combined systolic and diastolic congestive heart failure, NYHA class 2 and chronic kidney disease stage 3 (HCC/RAF) |
|  | 414 | Systolic heart failure secondary to coronary artery disease (HCC/RAF) |
|  | 414.8 | ACC/AHA stage C systolic heart failure due to ischemic cardiomyopathy (HCC/RAF) |
|  | 424.9 | Combined systolic and diastolic congestive heart failure due to valvular disease (HCC/RAF) |
|  | 425.X | Systolic heart failure secondary to hypertrophic obstructive cardiomyopathy (HCC/RAF) |
|  | 428.X | Acute systolic congestive heart failure (HCC/RAF) |
|  | 429.9 | Systolic heart failure, stage B (HCC/RAF) |
|  | 585.X | Benign hypertensive heart and kidney disease with NYHA class 2 systolic congestive heart failure and stage 1 chronic kidney disease (HCC/RAF) |
|  | V45.11 | Hypertensive heart and kidney disease with acute combined systolic and diastolic congestive heart failure and stage 5 chronic kidney disease on chronic dialysis (HCC/RAF) |
|  | 402.X | Accelerated hypertension with NYHA class 4 systolic heart failure (HCC/RAF) |
|  | 403 | Malignant systolic hypertension with CHF and chronic kidney disease (HCC/RAF) |
|  | 404.X | Malignant hypertensive heart and kidney disease with combined systolic and diastolic congestive heart failure, NYHA class 2 and chronic kidney disease stage 3 (HCC/RAF) |

*In cases where the same ICD diagnosis code included descriptions of both HFrEF and HFpEF diagnoses, each diagnosis description was independently evaluated and sorted into separate HFrEF and HFpEF diagnosis groups

Supplemental Table 3: Heart Failure with Mildy reduced Ejection Fraction Diagnoses

| **Diagnosis Code Type** | **Diagnosis Code** | **Diagnosis Description** |
| --- | --- | --- |
| ICD-10 | I50.30 | Heart failure with preserved ejection fraction, borderline, class I-IV |
|  | I50.30 | Heart failure with preserved ejection fraction, borderline, NYHA class I-IV |
|  | I50.22 | Heart failure with mildy reduced ejection fraction |
| ICD-9 | 428.9 | Heart failure with preserved ejection fraction, borderline, class I-IV |
|  | 428.9 | NYHA class 1-4 heart failure with borderline preserved ejection fraction |
|  | 428.22 | Heart failure with mildy reduced ejection fraction |

*Since HFmrEF does not have a unique ICD code, the diagnoses above were specifically selected and grouped into a separate HFmrEF category and excluded from HFrEF and HFpEF categories.

Supplemental Table 4: Heart Failure with Preserved Ejection Fraction Diagnoses

| **Diagnosis Code Type** | **Diagnosis Code** | **Diagnosis Description Example** |
| --- | --- | --- |
| ICD-10 | I11.0 | Malignant essential hypertension with congestive heart failure with preserved left ventricular function |
|  | I13.X | Hypertensive heart and chronic kidney disease with acute diastolic congestive heart failure |
|  | I25.10 | Heart failure, diastolic, due to CAD |
|  | I38 | Diastolic heart failure due to valvular disease |
|  | I50.1 | Left heart failure with left ejection fraction greater than or equal to 50 percent |
|  | I50.3X | Hypertensive heart disease with congestive heart failure with preserved left ventricular function |
|  | N18.X | Hypertensive heart disease with diastolic heart failure and stage 1 chronic kidney disease, unspecified HF chronicity |
|  | Z99.2 | Hypertensive heart disease with diastolic heart failure and end stage chronic kidney disease on dialysis |
| ICD-9 | 401 | Malignant essential hypertension with congestive heart failure with preserved left ventricular function (HCC/RAF) |
|  | 402.X | Malignant hypertensive heart disease with diastolic congestive heart failure, NYHA class 2 (HCC/RAF) |
|  | 404.X | Malignant hypertensive kidney and heart disease with congestive heart failure and preserved left ventricular function (HCC/RAF) |
|  | 414 | Diastolic heart failure secondary to coronary artery disease (HCC/RAF) |
|  | 424.9 | Diastolic heart failure due to valvular disease (HCC/RAF) |
|  | 425.X | Diastolic heart failure secondary to idiopathic cardiomyopathy, unspecified failure chronicity (HCC/RAF) |
|  | 428.X | Diastolic congestive heart failure, unspecified HF chronicity (HCC/RAF) |
|  | 429.9 | Diastolic heart failure, stage B (HCC/RAF) |
|  | 585.X | Hypertensive heart and kidney disease with chronic diastolic congestive heart failure and stage 1 chronic kidney disease (HCC/RAF) |
|  | V45.11 | Hypertensive heart and kidney disease with acute on chronic diastolic congestive heart failure and stage 5 chronic kidney disease on chronic dialysis (HCC/RAF) |

*In cases where the same ICD diagnosis code included descriptions of both HFrEF and HFpEF diagnoses, each diagnosis description was independently evaluated and sorted into separate HFrEF and HFpEF diagnosis groups

Supplemental Table 5: ASCVD Diagnoses

| **Diagnosis Code Type** | **Diagnosis Code** | **Diagnosis Description Example** |
| --- | --- | --- |
| ICD-10 |  |  |
|  |  |  |
|  | E08.5X | Diabetes mellitus due to underlying condition with diabetic peripheral angiopathy without gangrene (HCC/RAF) |
|  | E09.5X | Drug or chemical induced diabetes mellitus with diabetic peripheral angiopathy without gangrene |
|  | E10.5X | Type 1 diabetes with atherosclerosis of arteries of extremities (HCC/RAF) |
|  | E11.5X | Diabetes mellitus type 2 with atherosclerosis of arteries of extremities (HCC/RAF) |
|  | G45.X | Vertebral artery ischemia |
|  | G46.X | Posterior inferior cerebellar artery syndrome |
|  | I20.X | Unstable angina pectoris (HCC/RAF) |
|  | I21.X | ST elevation (STEMI) myocardial infarction of anterior wall. |
|  | I22.X | Subsequent myocardial infarction of anterior wall |
|  | I23.X | Hemopericardium after myocardial infarction (HCC/RAF) |
|  | I24.X | Occlusion of proximal portion of right coronary artery (HCC/RAF) |
|  | I25.X | Atherosclerosis of coronary artery with angina pectoris and documented spasm |
|  | I63.X | Thrombotic stroke involving precerebral artery (HCC/RAF) |
|  | I65.X | Basilar artery stenosis |
|  | I67.2 | Cerebrovascular disease, arteriosclerotic, post-stroke |
|  | I69.X | Aphasia following other cerebrovascular disease |
|  | I70.X | Aortic atherosclerosis, Peripheral arterial disease |
|  | Z86.73 | Chronic arterial ischemic stroke |
|  | Z95.3 | Presence of stent in coronary artery in patient with coronary artery disease |
|  | Z98.61 | Coronary arteriosclerosis after percutaneous coronary angioplasty |

Supplemental Table 6: Comparison of Mixed-Effect Model Results and Generalized Estimating Equation (GEE) Model Results

|  | Mixed-Effect Models | | GEEs | |
| --- | --- | --- | --- | --- |
| Measure | Monthly Post vs. Pre OR (95% CI) | P-value | Monthly Post vs. Pre OR (95% CI) | P-value |
| Composite GDMT for Heart Failure (at least one) | 1.035 (1.021, 1.049) | **<0.001** | 1.032 (0.996, 1.069) | 0.085 |
| Statin or PCSK9I | 1.006 (1.004, 1.008) | **<0.001** | 1.008 (1.000, 1.017) | 0.051 |
| Aspirin or P2Y12 Inhibitor | 0.988 (0.985, 0.992) | **<0.001** | 0.993 (0.985, 1.001) | 0.074 |
| Specified Beta Blocker | 1.015 (1.005, 1.025) | **0.003** | 1.012 (0.992, 1.033) | 0.252 |
| ACE, ARB or ARNI | 1.009 (1.000, 1.017) | **0.049** | 1.010 (0.994, 1.027) | 0.231 |
| MRA | 1.047 (1.039, 1.056) | **<0.001** | 1.039 (1.021, 1.058) | **<0.001** |
| Blood Pressure Control | 1.017 (1.015, 1.018) | **<0.001** | 1.016 (1.011, 1.021) | **<0.001** |

Results from the GEE models, presented alongside our primary mixed-effects models, showed comparable effect sizes for each metric. While a few effects that were statistically significant in the mixed-effects models were no longer statistically significant in the GEE models—likely due to the reduced efficiency of cluster-robust standard errors—the overall pattern of findings remained consistent.
